# A Model for Co-Designing Research With Older Adults: Findings From a Virtual Photovoice Study

**DOI:** 10.1093/geroni/igaf122.1061

**Published:** 2025-12-31

**Authors:** Joyce Weil

**Affiliations:** Towson University, Towson, Maryland, United States

## Abstract

Photovoice, designed as a collaborative process, offers ways to co-partner in research design with older adults. Findings from an Institutional Review Board-approved study where older adults who created photos about the meaning of home and neighborhood and were then interviewed via Zoom are used to illustrate the codesign process in a virtual photovoice study. Aging Patient-Centered Outcomes Learning Collaborative’s Older Adult Subcommittee, Healthier Black Elders’ Community Advisory Board, and older adults in the study, as three advisory groups, engaged in expanding the study’s reach, including groups of older persons, and in the study’s framing, conduction, and analysis. Data from 14 participants and a series of 20 selected photographs will be presented. These data will help illustrate the collaborative process used to create a model detailing the three co-design phases: codesign during the initial study design, throughout the study and data-collection process, and during dissemination. The model includes ways the researcher can reflect upon their role as subject and have older adults and other researchers navigate study protocols together as a form of empowerment. Findings include examples of partnering with older adults and conducting research with them, not just about them: e.g., the role of the researcher as a subject in their research, rethinking the photovoice study protocol and ways to capture desired photos within it, the role of humans in the data-collection process, and ways older adults took the study deeper in other written, narrative form and sent additional materials and research items to the researcher.

